# On-target action of anti-tropomyosin drugs regulates glucose metabolism

**DOI:** 10.1038/s41598-018-22946-x

**Published:** 2018-03-15

**Authors:** Anthony J. Kee, Jayshan Chagan, Jeng Yie Chan, Nicole S. Bryce, Christine A. Lucas, Jun Zeng, Jeff Hook, Herbert Treutlein, D. Ross Laybutt, Justine R. Stehn, Peter W. Gunning, Edna C. Hardeman

**Affiliations:** 1 School of Medical Sciences, UNSW Sydney, Sydney, NSW 2052 Australia; 2 Garvan Institute of Medical Research, St Vincent’s Hospital, UNSW Sydney, Sydney, NSW Australia; 3 MedChemSoft Solutions, Level 3 Brandon Park Drive, Wheelers Hill, 3150 VIC Australia; 4 Sanoosa Pty. Ltd., 35 Collins Street, Melbourne, 3000 VIC Australia; 5 Novogen Pty Ltd, 502/20 George St, Hornsby, NSW 2077 Australia

## Abstract

The development of novel small molecule inhibitors of the cancer-associated tropomyosin 3.1 (Tpm3.1) provides the ability to examine the metabolic function of specific actin filament populations. We have determined the ability of these anti-Tpm (ATM) compounds to regulate glucose metabolism in mice. Acute treatment (1 h) of wild-type (WT) mice with the compounds (TR100 and ATM1001) led to a decrease in glucose clearance due mainly to suppression of glucose-stimulated insulin secretion (GSIS) from the pancreatic islets. The impact of the drugs on GSIS was significantly less in Tpm3.1 knock out (KO) mice indicating that the drug action is on-target. Experiments in MIN6 β-cells indicated that the inhibition of GSIS by the drugs was due to disruption to the cortical actin cytoskeleton. The impact of the drugs on insulin-stimulated glucose uptake (ISGU) was also examined in skeletal muscle *ex vivo*. In the absence of drug, ISGU was decreased in KO compared to WT muscle, confirming a role of Tpm3.1 in glucose uptake. Both compounds suppressed ISGU in WT muscle, but in the KO muscle there was little impact of the drugs. Collectively, this data indicates that the ATM drugs affect glucose metabolism *in vivo* by inhibiting Tpm3.1’s function with few off-target effects.

## Introduction

As well as forming part of the striated muscle contractile apparatus, the actin cytoskeleton is involved in many fundamental processes in all eukaryotic cells including cell proliferation, cell migration, cell adhesion and intracellular trafficking. The high sequence homology between the 6 actin isoforms^1^ has made it difficult to design compounds that discriminate between the different actin filaments in cells based on their actin composition^2,3^.

The discovery that the tropomyosins (Tpms), which form co-polymers with actin, define the intrinsic functional capacity of the filament has provided the ability to discriminate between different filament populations^4^. Tpms are encoded by four genes and many isoforms (n > 40) arise from these genes by exon splicing and alternate promoter usage^1^. Three isoforms are striated muscle-specific and form part of the actin thin filament of the contractile apparatus where they regulate actin-myosin interactions and give strength and stability to the contractile apparatus^5^. The other Tpm isoforms are considered to be non-muscle or cytoskeletal. Previous studies have demonstrated that both Tpm and actin isoforms segregate into functionally distinct filament populations in different cell types^6–16^. Tpms are now viewed as the ‘gate keepers of the actin cytoskeleton’^17^, regulating the interaction of other actin regulatory proteins with the actin filament. This provides a means to independently regulate the cytoskeleton at different sites within the cell and to tailor the function of actin filaments at these different sites. This also provides the opportunity to target distinct actin filament populations in cells based on Tpm composition.

Studies using gene knock-out and overexpressing transgenic approaches have demonstrated that Tpms in an isoform-specific manner regulate a number of specific physiological processes in yeast, insect and mammalian systems^4,18,19^. Tpm3.1 has been shown to be essential for embryonic stem cell proliferation^20^, to regulate organ size and cell proliferation in mice^10^ and is required for cancer cell survival^21^. Cell transformation is accompanied by changes in the Tpm isoform composition of their actin cytoskeleton^4^ with Tpm3.1 and Tpm4.2 consistently retained by all human cancer cells thus far examined^21^. Tpm4.2 has also been shown to have an important role in the terminal stages of platelet production^22^. In two recent studies, Tpm3.1-containing actin filaments have also been shown to regulate glucose uptake in mice in an isoform-specific manner^23,24^. In the first study, insulin stimulation of Akt resulted in phosphorylation of Tmod3, capping Tpm3.1-containing actin filaments that are involved in actin reorganization at the cell periphery and increased incorporation of the GLUT4 glucose transporter into the plasma membrane (PM)^24^. In the other study, Tpm3.1 was shown to regulate insulin-stimulated glucose uptake in mouse skeletal muscle and adipose tissue^23^. Evidence was also provided for two populations of actin filaments required for GLUT4 vesicle trafficking, one containing Tpm3.1 and MyoIIA motors, and the other lacking Tpm, but interacting with Myo1c^23^.

We have developed compounds that target Tpm3.1 as anti-cancer agents^21,25^. These anti-Tpm (ATM) compounds, disrupt Tpm3.1-containing actin filaments in cancer and non-cancer cells^21,23,25,26^, are anti-proliferative and have anti-tumor activity in mouse xenograph models^21,25^. *In vitro* experiments using purified proteins showed that these compounds disrupt Tpm3.1-regulated actin filament dynamics by increasing the rate of filament depolymerisation^21,25,26^. In the present study, we show that the ATM compounds impacted two processes known to be regulated by the actin cytoskeleton, insulin-stimulated glucose uptake and insulin secretion^27,28^. Comparison of the effect of the compounds in wild-type (WT) versus Tpm3.1 knock-out (KO) mice indicate that the compounds are operating by specifically inhibiting Tpm3.1 function.

## Results

### Strategy to assess on- and off-target impact of the ATM compounds

The major objective of this study was to establish if ATM drugs can specifically target the role of Tpm3.1 in glucose metabolism as we had previously shown that a major phenotype in Tpm3.1 transgenic and knock-out (KO) mice was altered glucose uptake^23^. The on- and off-target impact of the compounds were evaluated by comparing the impact of the drugs in wild-type versus Tpm3.1 KO mice. Confirmation of an on-target effect was shown if there was no impact of the drug in KO mice, and the response to the drug in the WT mice mimicked the vehicle-treated KO mice.

### ATM-1001 structure and impact on Tpm3.1 filaments

**Figure 1:**
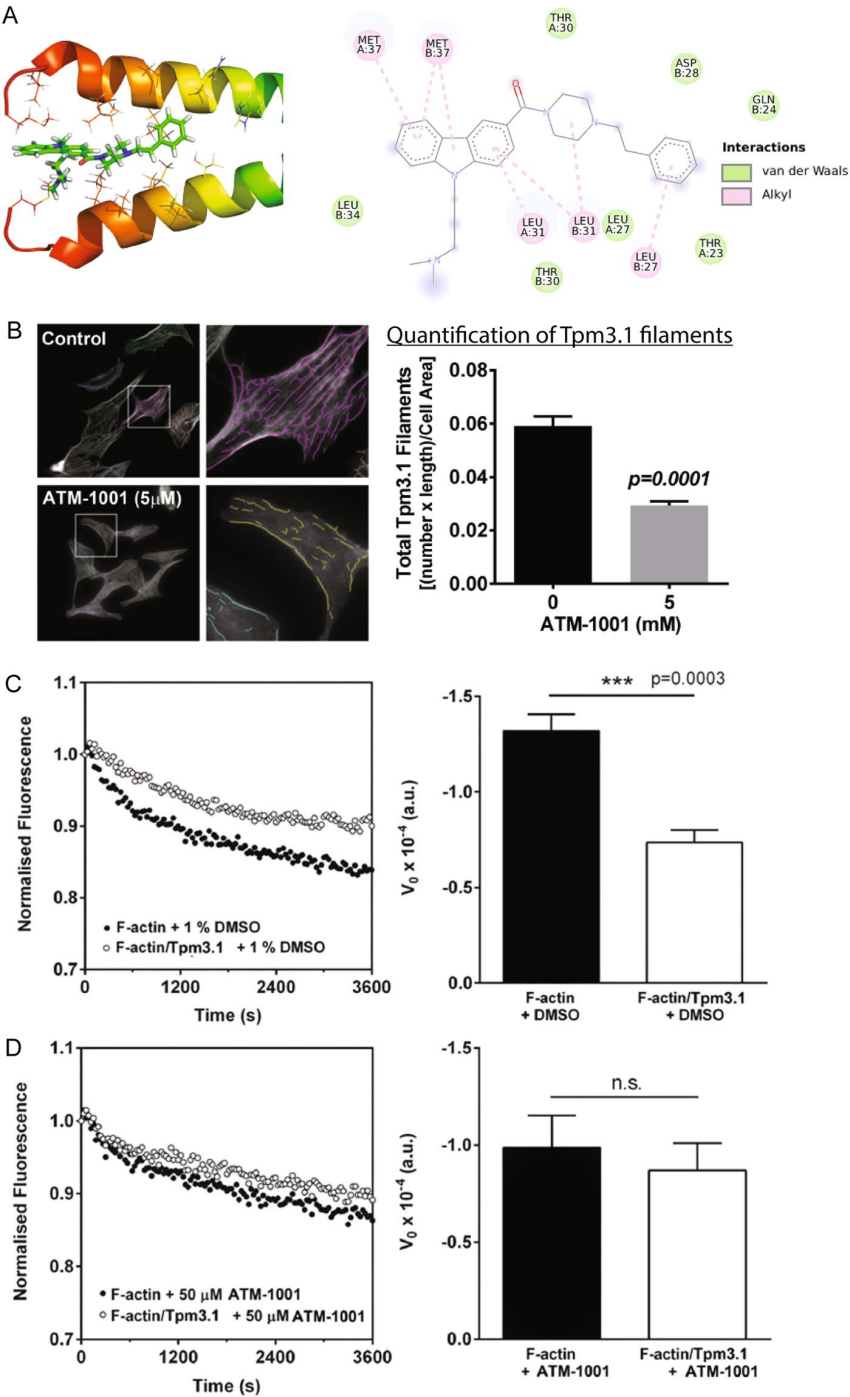
ATM-1001 targets Tpm3.1-containing actin filaments. (**A**) Structural diagram of the predicted molecular interactions of ATM-1001 and the C-terminus (last 37 amino acids) of the two Tpm3.1 helices. (**B**) ATM-1001 impact on Tpm3.1 filaments was quantitated in MEF cells using a High Content Imaging assay in combination with the CSIRO linear feature detection algorithm. Tpm3.1 filaments/cell area were quantitated using >300 cells (n = 3 independent experiments; ****p < 0.0001, T-test). (**C**) Depolymerization time course of actin filaments (35% pyrene labeled) with or without saturating amounts of Tpm3.1. Data: mean ± SEM, 4–6 replicates (***p < 0.001, T-test). (**D**) Tpm3.1 was pre-incubated with 50 μM ATM-1001 or 1% (v/v) DMSO prior to mixing with F-actin. Fluorescence data normalized to the T0 fluorescence and initial rates of depolymerisation (V0), calculated from the first 600 s, for F-actin alone or Tpm3.1/F-actin in the presence of DMSO or ATM-1001.

The impact of two ATM compounds was examined, our first-in-class compound, TR100^21^ and a novel compound, ATM-1001 which was identified from a previously described library of compounds on the basis of its cytotoxic potency and ability to disrupt Tpm3.1-containing microfilaments^25^. Molecular modelling of ATM1001 with a peptide containing the last 37 amino acids of Tpm3.1 shows that the scaffold of ATM-1001 fits into the cavity between two helixes of Tpm showing close contacts with Leu31 and Met37 of the C-terminal peptide (corresponding to Leu243 and Met248 in the full-length Tpm3.1 protein) (Fig. 1A). The trimethylammonium [N-Me3(+)] charged group of ATM1001 interacts with the Tpm C-terminus through electrostatic interactions, while the phenyl ring provides hydrophobic interactions with Leu27. Using a High Content Imaging (HCI) assay, we found that Tpm3.1-containing filament bundles in MEF cells were disrupted by 5 μM ATM-1001 (Fig. 1B) and showed that ATM-1001 inhibits Tpm3.1’s ability to protect actin from depolymerization by quantifying polymerized actin over time (Fig. 1C,D). These activities are similar to that reported for other anti-tropomyosin (ATM) compounds (TR100 and ATM-3507)^21,25,26^.

### ATM-1001 plasma clearance and tissue distribution

We examined the plasma clearance and distribution of ATM-1001 into metabolically active tissues following a single bolus injection of the compound (40 mg/kg body weight) in wild-type and Tpm3.1 KO mice. ATM-1001 was cleared rapidly from the blood and reached a maximum concentration in the tissues (skeletal muscle and pancreas) at 1–2 h after injection (Figure S1). Interestingly, the drug concentration in pancreas was higher than muscle, perhaps reflecting a greater blood flow to the visceral tissues compared to muscle. Importantly, the plasma clearance and tissue distribution of ATM-1001 were similar in WT and KO mice (Figure S1) indicating that Tpm3.1 is not involved in drug clearance and tissue distribution.

### ATM drugs alter glucose clearance via impacts on Tpm3.1

**Figure 2:**
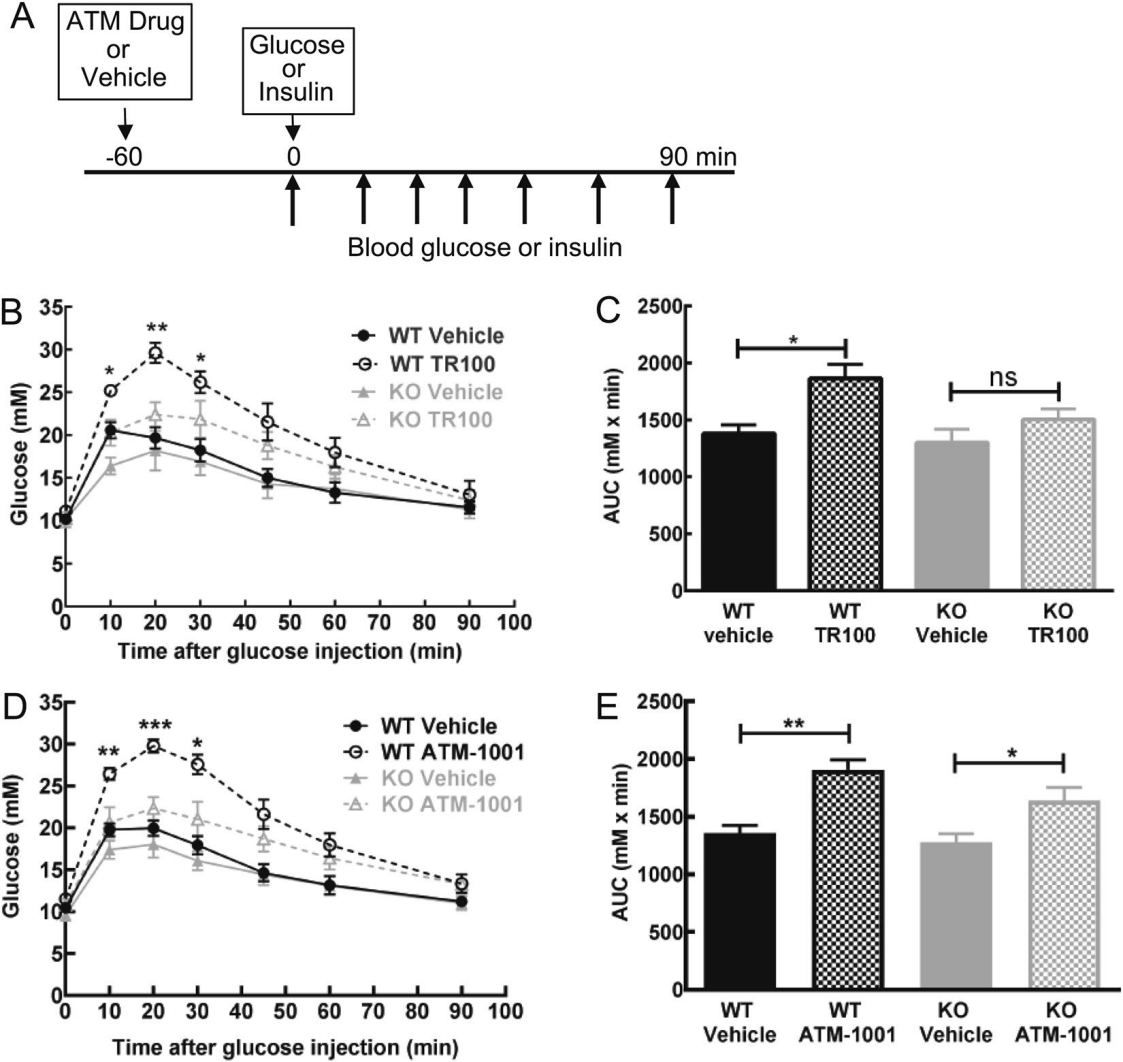
Anti-tropomyosin drugs have on-target impact on glucose clearance. (**A**) Experimental design to examine acute (single dose) impact of ATM drugs on glucose metabolism. (**B**) Glucose tolerance test (GTT; 1.5 g glucose/kg BW, *i.p*.) and (**C**) area-under-the-curve for the GTT, 1 h after TR100 (40 mg/kg BW, *i.p*.) or vehicle injection in wild-type (WT) and Tpm3.1 knock-out (KO) mice (n = 5/group). (**D**) Glucose tolerance test and (**E**) area-under-the-curve for the GTT, 1 h after ATM-1001 (40 mg/kg BW, *i.p*.) or vehicle injection in wild-type and Tpm3.1 KO mice (n = 6–7/group). Results are shown as mean ± SEM and the results of statistical analysis (ANOVA with post-hoc Tukey’s multiple comparison test) are indicated: ns P > 0.05, *P < 0.05, **P < 0.01, ***P < 0.001.

To assess the impact of the ATM compounds on glucose metabolism we first confirmed that injection of the vehicle alone had no impact on glucose clearance compared to saline-injected controls (Figure S2A). To provide an initial assessment of the impact of the ATM compounds on glucose metabolism, ATM drugs (40 mg/kg BW, *i.p*.) and vehicle (Dexolve-7) were administered to the Tpm3.1 WT and KO mice and 1 h later an intraperitoneal glucose tolerance test was performed (see Fig. 2A for study design). One hour following TR100 or ATM-1001 injection blood glucose was similar to vehicle-injected controls (Figure S3A,C). However, both TR100 and ATM-1001 significantly decreased glucose clearance in WT and KO mice (Fig. 2B–E). Importantly, the impact of the drugs on clearance was significantly less in the KO versus the WT mice for both TR100 and ATM-1001 (Fig. 2C and E, respectively) indicating in the WT mice the drugs impacted on Tpm3.1 to decrease glucose clearance. The impact of the drugs on glucose clearance was relatively short lived, at 8 h after drug administration there was little impact on glucose clearance (Figure S4). This is presumably due to low drug concentration in plasma and tissue at this time after administration (Figure S1).

### The ATM drugs do not alter whole body insulin sensitivity but inhibit insulin-stimulated glucose uptake in skeletal muscle *ex vivo*

**Figure 3:**
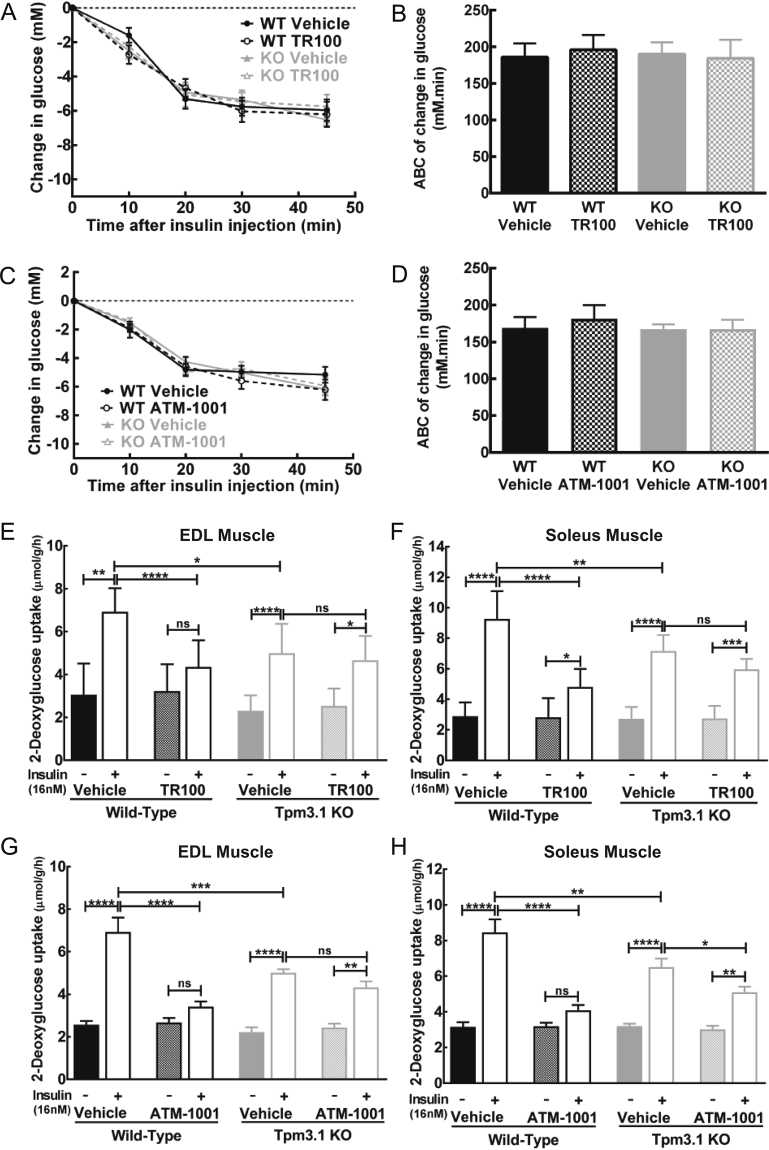
ATM drugs do not impact on whole body insulin sensitivity, but inhibit insulin-stimulated glucose uptake in skeletal muscle *ex vivo*. (**A**) Insulin tolerance test (ITT; 2U insulin/kg BW, *i.p*.) and (**B**) area-above-the-curve for the ITT, 1 h after TR100 (40 mg/kg BW, *i.p*.) or vehicle injection in wild-type (WT) and Tpm3.1 knock-out (KO) mice. (**C**) Insulin tolerance test and (**D**) area-above-the-curve for the ITT, 1 h after ATM-1001 (40 mg/kg BW, *i.p*.) or vehicle injection in WT and Tpm3.1 KO mice (n = 5–6/group). (**E**,**F**) Impact of TR100 (50 μM) or vehicle on insulin-stimulated glucose uptake in WT and Tpm3.1 KO (**E**) extensor digitorum longus (EDL) and (**F**) soleus muscle. (**G**,**H**) Impact of ATM-1001 (50 μM) or vehicle on insulin-stimulated glucose uptake in WT and Tpm3.1 KO (**G**) EDL and (**H**) soleus muscle. Results are shown as mean ± SEM (*n* = 6–8/group) and the results of statistical analysis (ANOVA with post-hoc Tukey’s multiple comparison test) are indicated: ns > 0.05, *P < 0.05, **P < 0.01, ***P < 0.001, ****P < 0.0001.

An insulin tolerance test was then performed following ATM drug administration to examine whether the altered glucose clearance with the ATM drugs is due to impacts on whole-body insulin sensitivity. The clearance of glucose in response to insulin was similar in vehicle-treated WT and KO mice and both ATM drugs (TR100 and ATM-1001) had no impact on insulin-stimulated glucose clearance (Fig. 3).

The lack of impact of the ATMs on insulin-stimulated glucose clearance was surprising as we had previously shown that Tpm3.1 regulated insulin-stimulated glucose uptake (ISGU) in muscle and adipose tissue, and TR100 inhibited this process in 3T3-L1 adipocytes^23^. We therefore determined whether the ATM drugs impacted directly on ISGU in skeletal muscle, the major tissue responsible for glucose disposal. Extensor digitorum longus (EDL; fast-twitch, glycolytic) and soleus (slow-twitch, oxidative) muscles were removed from WT and KO mice and incubated with vehicle or ATM drugs and ISGU measured. In the absence of insulin, glucose uptake in vehicle-treated WT and KO were similar and there was little impact of the ATM drugs indicating that Tpm3.1 does not regulate basal glucose uptake (Fig. 3E–H). In contrast, in response to insulin the rate of glucose uptake was decreased in vehicle-treated KO compared to vehicle-treated WT muscles (both EDL and soleus) (Fig. 3E–H), confirming a role for Tpm3.1 in regulating ISGU.

Initially, we examined the impact of the ATM drugs on ISGU at 25 µM, an equivalent concentration to that found in muscle (25–30 µmol/kg) after drug administration (40 mg/kg BW) to the mice (Figure S1B), and found no significant impact on glucose uptake (15% and 17% inhibition of ISGU in EDL and soleus muscle, respectively at P = 0.093). This provides a potential explanation for the lack of an effect of the ATM compounds on insulin-stimulated glucose clearance (Fig. 3A–D). At drug concentration of 50 µM, however both TR100 and ATM-1001 significantly inhibited ISGU in WT EDL and soleus muscles (Fig. 3E–H). Importantly, at this concentration the ATM compounds had lesser impact on ISGU in the KO compared to WT muscles (Fig. 3E–H). Collectively, these data indicate that these ATM drugs inhibit ISGU through impacts on Tpm3.1.

**Figure 4:**
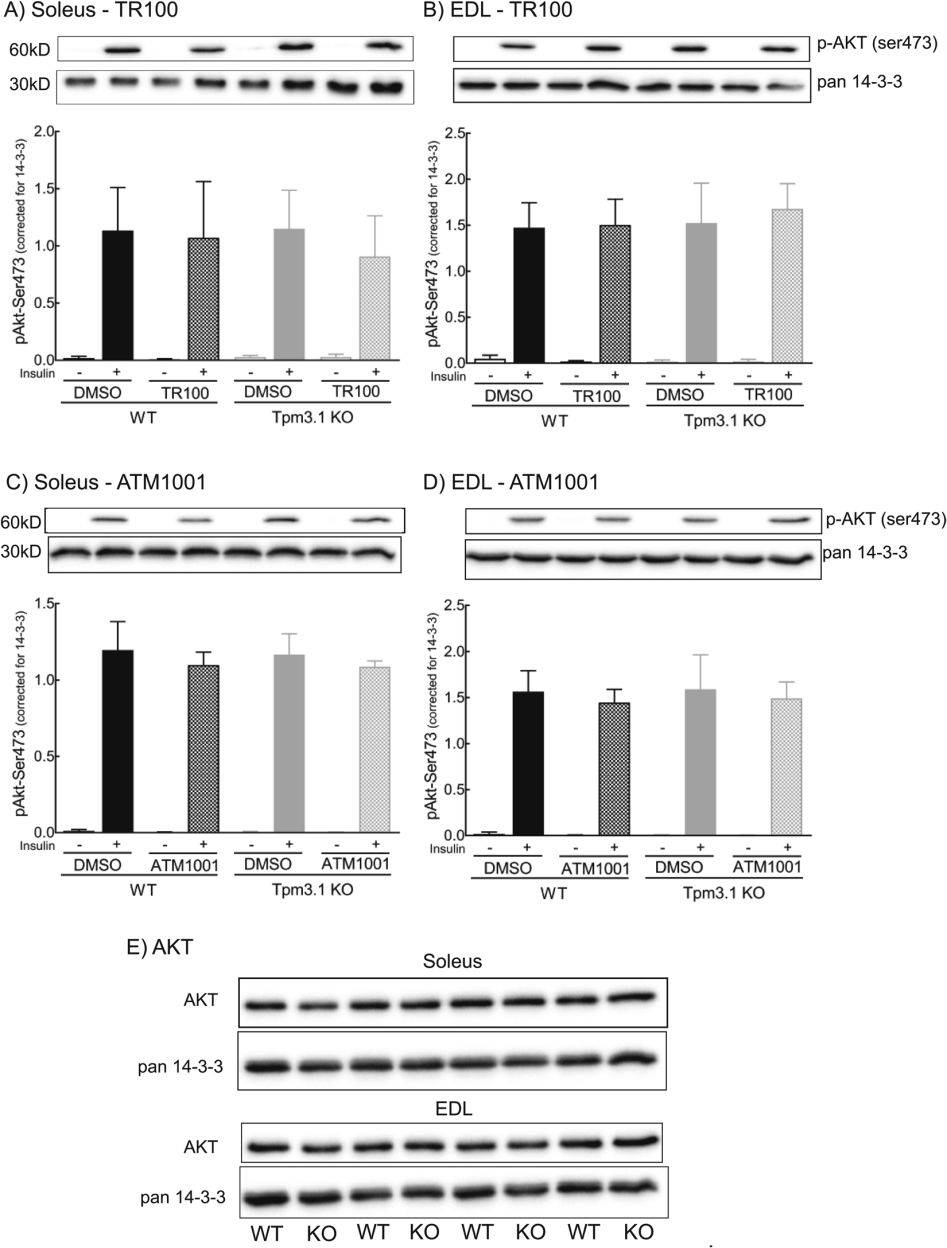
ATM compounds do not impact on insulin-stimulated Akt phosphorylation. Impact of 50 μM TR100 (**A**,**B**) and 50 μM ATM-1001 (**C**,**D**) on *ex vivo* insulin-stimulated phosphorylation of Akt (Ser473) in soleus (**A**,**C**) and extensor digitorum longus (EDL) muscle (**B**,**D**) from wild-type (WT) and Tpm3.1 knock-out (KO) mice. Representative blots of P-Akt and the house-keeping loading control protein, 14-3-3 are shown (same samples run on separate gels for Akt and 14-3-3) and as well as the quantitation of P-Akt for *n* = 5–7 mice per treatment group. Data are represented as mean ± SEM. Densitometry values on each gel were corrected for loading (pan-14-3-3) and then results are expressed as a ratio to an internal standard (insulin-stimulated muscle) run on each gel. There was a similar effect of insulin on Akt phosphorylation in both muscles from WT and Tpm3.1 KO mice with and without ATM compound (1-way ANOVA). (**E**) Western blots of total AKT in WT and Tpm3.1 KO Soleus and EDL muscle. Total AKT levels were similar in WT and Tpm3.1 KO soleus and EDL muscle. The blots for Akt and 14-3-3 were from the same samples run on separate gels.

The Akt pathway has been identified as the most important insulin signalling pathway involved in ISGU^29^. To investigate whether the inhibition of glucose uptake with the ATM compounds is via impacts on proximal insulin-signalling, we examined insulin-stimulated Akt phosphorylation (Ser473) in ATM-treated (TR100 and ATM-1001, 50 μM) muscle collected from the same *ex vivo* incubations used for glucose uptake measurements. Western densitometry showed that total AKT levels in the basal state and insulin-stimulated Akt-Ser473 phosphorylation was similar in vehicle-treated WT versus Tpm3.1 KO muscle (Fig. 4E). Similarly, TR100 and ATM-1001 had no impact on insulin-stimulated Akt phosphorylation in WT and KO muscles (Fig. 4A–D). These data confirm previous reports that Tpm3.1 does not regulate proximal insulin signalling^23^ and indicates that the ATM drugs impact on glucose uptake “down-stream” of Akt signalling.

### ATM drugs alter glucose-stimulated insulin secretion via impacts on Tpm3.1

**Figure 5:**
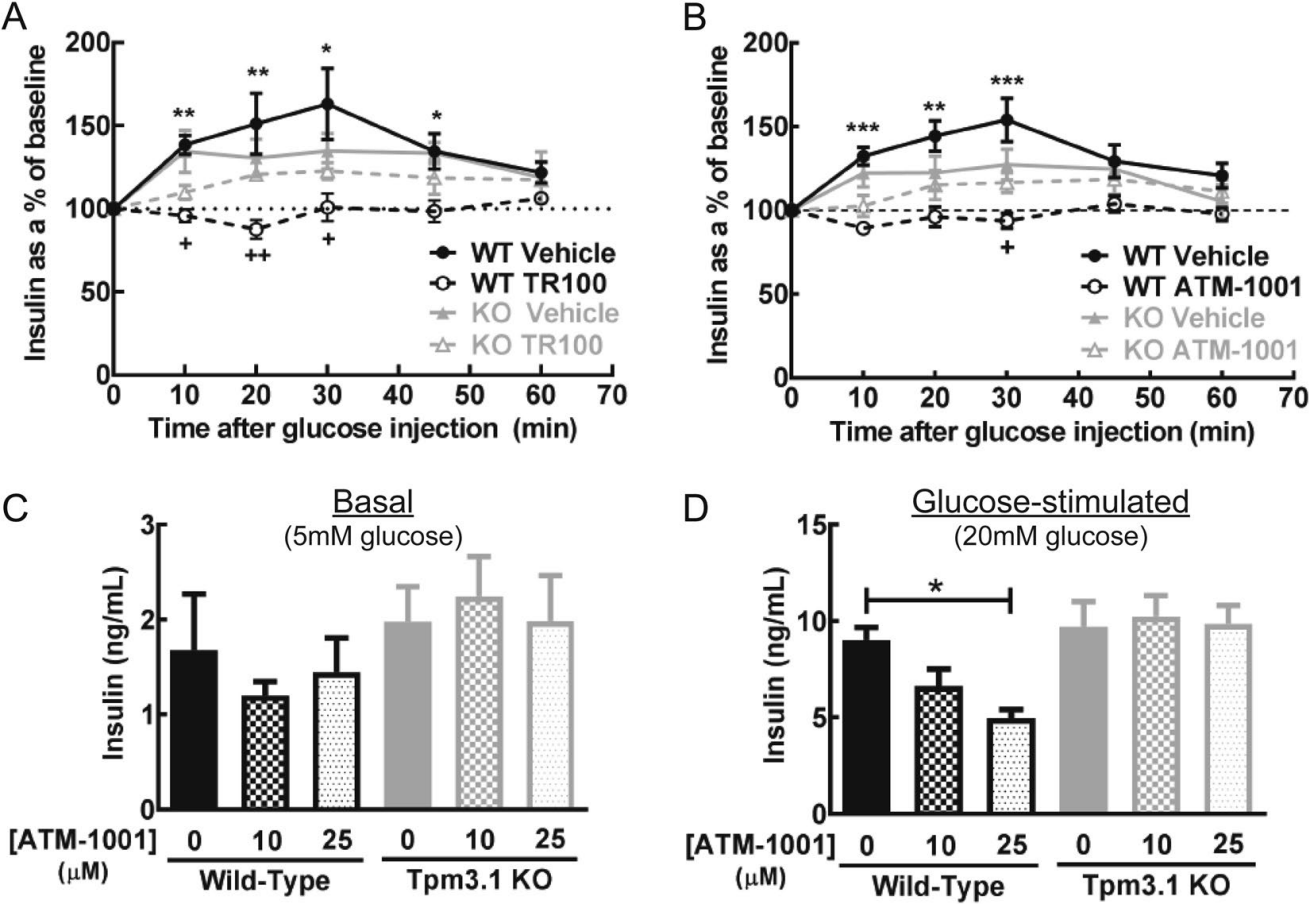
ATM drugs impact on insulin secretion. (**A** and **B**) Changes in insulin levels during a glucose tolerance test (2 g glucose/kg BW, *i.p*.) in wild-type (WT) and Tpm3.1 knock-out (KO) mice (n = 5/group), 1 h after (**A**) TR100, (**B**) ATM-1001 (40 mg/kg BW, *i.p*.) or vehicle administration. Results of statistical analysis (ANOVA with post-hoc Tukey’s multiple comparison test) are indicated by: (1) *P < 0.05; **P < 0.01; ***P < 0.001, for drug- versus vehicle-treated mice of the same genotype; and (2) ^+^P < 0.05; ^++^P < 0.01, for drug-treated WT versus drug-treated KO mice. (**C** and **D**) Insulin secretion in pancreatic islets isolated from WT and Tpm3.1 KO mice treated with increasing amounts of ATM-1001 (1 h treatment). There was no impact of ATM-1001 on (**C**) basal (5 mM glucose) secretion, but there was a dose-dependent suppression of (**D**) glucose-stimulated (20 mM glucose) insulin secretion with increasing concentration of drug in WT but not KO islets. Results are mean ± SEM from n = 5 islets/group. The results of statistical analysis (ANOVA with post-hoc Tukey’s multiple comparison test) are indicated: *P < 0.05; **P < 0.01, ***P < 0.001.

That the ATM drugs decreased glucose clearance without impacting on whole body insulin sensitivity (Fig. 3A,B) raised the possibility that the drugs altered glucose-stimulated insulin secretion (GSIS). To examine this, ATM drugs were administered and 1 h later a GTT was performed and insulin levels measured (see Fig. 2A for study design). Injection of the drug-vehicle alone had no impact on glucose-stimulated insulin secretion compared to saline-injected control mice (Figure S2B). In the basal state (prior to glucose injection), ATM drugs had no impact on insulin levels compared to vehicle-treated WT and KO mice (Figure S3B,D). In vehicle-treated mice, there was a trend towards a smaller increase in insulin levels following glucose injection in KO versus WT mice (Fig. 5A,B), but the difference was not statistically significant (AUC of % change in insulin: WT-vehicle = 8205 ± 260%.min, KO-vehicle = 7466 ± 266%.min; P = 0.08). Both TR100 and ATM-1001 completely suppressed insulin secretion in the WT mice. In these mice, insulin levels were not significantly different from baseline at all time points after glucose injection (Fig. 5A,B). In contrast, in the KO mice glucose-simulated insulin secretion was equivalent in vehicle and ATM treated mice (Fig. 5A,B). This indicates that the inhibition of GSIS by the ATM compounds in the WT mice was mainly through impacts on Tpm3.1.

To confirm an impact of the ATM drugs on insulin secretion, we examined the effect of the ATM-1001 on insulin secretion in pancreatic islets from the WT and KO mice. Islets were incubated with ATM-1001 for 1 h and insulin secretion (into the media) was measured in presence of basal (5 mM) and elevated glucose (20 mM). Basal and glucose-stimulated insulin secretion (GSIS) was similar in vehicle-treated WT and KO islets (Fig. 5C,D). ATM-1001 had no significant impact on insulin secretion in basal conditions (Fig. 5C), in agreement with lack of impact on fasting insulin in the mice (Figure S3D). However, in the WT islets there was a dose-dependent decrease in GSIS with ATM-1001 treatment, whereas in the KO islets ATM-1001 had no effect on GSIS (Fig. 5D). Collectively, these data indicate that ATM-1001 is impacting on Tpm3.1 to decrease GSIS.

### ATM drugs alter insulin secretion by disrupting cortical actin filaments

**Figure 6:**
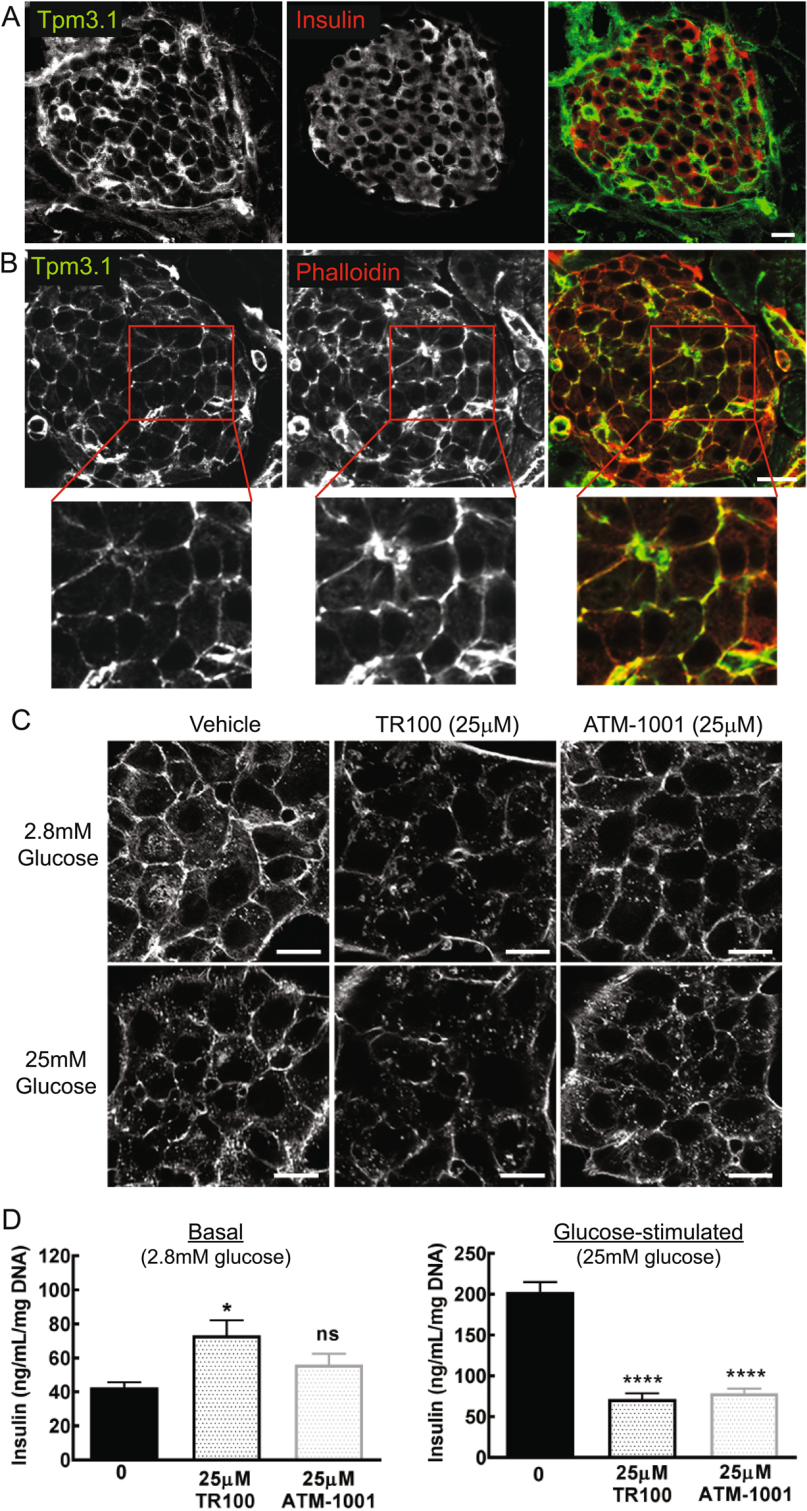
Tpm3.1 is at the cell cortex with actin and ATM drugs impact on insulin secretion by disrupting cortical actin filaments in β cells. (**A**) Representative immunofluorescent confocal images of Tpm3.1 (γ/9d antibody) and insulin and (**B**) Tpm3.1 and filamentous actin (F-actin; Phalloidin stain) in wild-type mouse pancreas. Tpm3.1 is concentrated at the cell cortex in β cells (upper images) colocalized with F-actin (magnified insets in **B**). Scale bars = 20 μm. (**C**) Representative confocal images of MIN-6 cells at the cell centre, treated with vehicle, TR100 or ATM-1001 (1 h) in the presence of with basal (2.8 mM) or high (25 mM) glucose and stained for F-actin (Phalloidin) after 20 min glucose incubation. In vehicle-treated cells, there was a loss of F-actin at the cell cortex in cells in high versus basal glucose. TR100 and ATM-1001 disrupted actin at the cell cortex in both basal and high glucose conditions. (**D**) Insulin secretion in MIN6 β cells treated with increasing amounts of TR100 and ATM-1001. Cells were incubated with basal (2.8 mM) or high (25 mM) glucose in the presence of vehicle or ATM drug and insulin secretion measured over a 1 h period. There was a significant increase in basal secretion with TR100, but not ATM-1001 treatment. Both TR100 and ATM-1001 suppressed glucose-stimulated insulin secretion. Results are shown as mean ± SEM for n = 5–6 independent experiments. Statistical analysis: ANOVA with post-hoc Tukey’s multiple comparison test (ns P > 0.05, *P < 0.05). Scale bars = 20 µM.

Actin plays a major role in insulin granule exocytosis in regulating the trafficking and/or insertion of the granules into the plasma membrane^27^. Treatment of β-cells with actin-destabilizing drugs (latrunculin) has been shown to increase insulin secretion^30–32^ leading to the hypothesis that the actin cytoskeleton acts as a barrier for granule exocytosis. We therefore have examined whether the impact of the ATM drugs on insulin secretion is due to alteration to the cortical actin cytoskeleton. We first demonstrate that the drug target, Tpm3.1 is present at the cell periphery in β-cells (stained with insulin) of mouse pancreas (Fig. 6A) in close association with F-actin (Phalloidin staining) (Fig. 6B) as has been observed for Tpm3.1 in other mouse tissues^23^.

We examined the impact of the ATM drugs on glucose-stimulated actin remodelling in MIN-6 β-cells. In the basal state (2.8 mM glucose), F-actin was concentrated at the cell cortex in vehicle-treated cells (Fig. 5C). As previously reported for MIN-6 and other β-cell lines^30–32^, with glucose stimulation (25 mM) in vehicle-treated cells there was significant disruption of F-actin at the cell cortex (Fig. 6C) and an increase in small F-actin puncta in the cell interior, which was associated with a substantial (4-fold) increase in insulin secretion (Fig. 6D). Treatment of cells with TR100 and ATM-1001 led to a significant loss of F-actin at the cell cortex in both the basal and glucose-stimulated states (Fig. 6C). This was associated with increased insulin secretion in the basal state (although only statistically significant for TR100) and a suppression of glucose-stimulated glucose secretion (Fig. 6D). Collectively, this data suggests that the ATM compounds inhibit insulin secretion via impacting on cortical actin filaments.

## Discussion

Existing anti-actin drugs (e.g., cytochalasin and latrunculin) have significant cardiac and respiratory muscle toxicity due to their inability to discriminate between muscle and non-muscle actin isoforms, which limits their *in vivo* use as anti-cancer agents^2,3^. We have therefore developed novel small molecule inhibitors of tropomyosin that are able to target specific actin filament populations^21,25,26^ without impacting on cardiac muscle function^21^. These ATM compounds are able to disable the cancer-associated Tpm3.1/actin filament system and have potent synergy with current anti-cancer drugs in animal xenograph models^21,25^. In the present study, we show that ATM compounds are also able to target Tpm-regulated metabolic processes in mice. This includes insulin-stimulated glucose uptake in mouse skeletal muscle and glucose-stimulated insulin secretion *in vivo* in the mouse and *ex vivo* in pancreatic islets. We show that these effects of the ATM compounds are largely due to on-target impacts on the Tpm3.1 isoform. Our finding predicts that use of ATMs as anti-cancer agents will elicit mild diabetic features that will persist during the course of treatment, but will reverse once treatment ends.

Previously we demonstrated that Tpm3.1 regulates insulin-stimulated glucose uptake (ISGU) in skeletal muscle and adipose tissue^23^ and here we confirm these findings by showing reduced ISGU in the vehicle-treated muscle from Tpm3.1 KO compared to WT mice (Fig. 3E–H). This provides the ideal system to test the ability of the ATM drugs to specifically target a Tpm3.1-regulated physiological process. Incubation of WT skeletal muscle with either TR100 or ATM-1001 led to a very robust suppression of ISGU while in the KO muscle the ATM compounds had no significant impact. This represents a very clear demonstration that these compounds inhibit ISGU via an on-target impact on Tpm3.1 function. Furthermore, as the drugs had little impact on ISGU in KO muscles. We conclude that for glucose uptake there is little off-target impacts of the ATM compounds. In addition, that the impact of the drugs on ISGU in the WT muscle was greater than the response to the absence of Tpm3.1 in the KO (untreated KO vs untreated WT) suggests that there has been compensation in the KO animal to limit defects in ISGU which is not possible when Tpm3.1 function is acutely inhibited with the ATM compounds. The compensation is unlikely to be by other Tpm isoforms as expression of other Tpms in the Tpm3.1 KO skeletal muscle are unchanged^23^, but maybe be due to changes in other components of the actin cytoskeleton involved in the process of glucose uptake.

The main signalling pathway responsible for insulin-dependent glucose uptake in skeletal muscle and adipose tissue is the Akt pathway^33,34^. This is mediated by an Akt-dependent increase in translocation and insertion of GLUT4-containing vesicles into the surface membranes (plasma and transverse tubule membranes in skeletal muscle). Recently, a number of down-stream targets of Akt have been identified that mediate aspects of GLUT4 exocytosis^34^. This includes molecules involved in regulating the release of GLUT4 vesicles from the intracellular storage compartment (TBC1D1 and TBC1D4)^28,29^, as well as components of the actin cytoskeleton^24^. One such direct target of Akt is the actin filament capping protein tropomodulin 3 (Tmod3). The insulin-dependent Akt phosphorylation of Tmod3 leads to capping of Tpm3.1-containing actin filaments which has been shown to be required for stimulation of GLUT4 vesicle exocytosis and glucose uptake^24^. The lack of an effect of the ATM compounds on insulin-stimulated Akt phosphorylation indicate that these compounds act down-stream of Akt to effect glucose uptake presumably acting specifically on the Tpm3.1 filament population involved in regulating GLUT4 exocytosis^23^.

In addition to glucose uptake the ATM compounds have a potent impact on regulated insulin secretion. In WT mice *in vivo* and in isolated islets from the mice, both ATM drugs inhibited insulin secretion in response to glucose stimulation. In a similar fashion to that observed for glucose uptake in skeletal muscle, the drugs had no significant impact on GSIS in islets from the KO mice. This demonstrates that for insulin secretion the drugs inhibitory effect is via impacts on Tpm3.1 and that there are few off-target effects of the drugs. Furthermore, in the untreated KO and WT mice, insulin secretion *in vivo* and in the islets were similar, suggesting there was compensation for the loss of Tpm3.1 to normalise GSIS which does not occur when Tpm3.1 is inhibited acutely with the ATM compounds. Collectively, these findings indicate that Tpm3.1 may also play a role in regulated insulin secretion.

Actin has been implicated in multiple steps of the insulin secretory pathway^27^. In beta cells, stimulation with high glucose induces remodelling of focal adhesions and activation of cytoskeletal adaptors which leads to insulin granule movement along actin filament tracks assisted by myosin motors (MyoVa, II) allowing the approach to fusion machinery at the cell membrane^35^. F-actin and Myosin II motors are also recruited to coat the surface of the granules during fusion with the plasma membrane to stabilise the shape of secretory granules, maintain the opening of the fusion pore and to power the discharge of granule contents^36–39^. Cortical actin remodelling and the breakdown of the dense cortical actin mesh, which was observed in this study in MIN6 cells (Fig. 5C), is also thought to allow granule approach to the membrane^30–32^. In the present study we show that Tpm3.1 is associated with the cortical actin network in pancreatic β-cells (Fig. 5A) and the ATM compounds are able to breakdown this peripheral actin network leading to an increase in insulin secretion in MIN6 cells (Fig. 4C). This is similar to what has been observed with anti-actin drugs, that disrupt F-actin (e.g., cytochalasins) or prevent new filament formation (e.g., latrunculin). These data are consistent with Tpm3.1-containing actin filaments being involved in the remodelling of the cortical actin network during regulated secretion.

There exists a diverse range of non-muscle Tpm isoforms, due to extensive alternate splicing of the four Tpm genes^4^. ATM compounds were designed to recognise a region coded by the 9d exon of the Tpm3 gene^21,25^ that has some sequence conservation across the four Tpm genes^40^. Therefore, drugs designed to target Tpm3.1 may target other isoforms of Tpm albeit with lesser efficiency^21^. However, that KO mice were completely resistant to the impacts of the drugs on ISGU and GSIS suggests that if these drugs are targeting other Tpms they are not involved in these processes. This highlights the ability to target specific actin regulated processes via manipulating Tpm function.

It is well established that Tpms form a continuous polymer through interaction between the C-terminus and N-terminus region of the Tpm dimers^4,41^. Individually a Tpm dimer has a low affinity to interact with the actin filament, however collectively the Tpm polymer is able to associate along the entire length of the actin filament providing a stable interaction^4,41^. ATM compounds ATM-1001 and TR100 were designed to target the C-terminus region of Tpm3.1 (exon9d), and it appears that these compounds bind Tpm dimers and become incorporated within the growing actin filament^26^. The presence of ATMs bound to the Tpm polymer on the actin filament has been shown to inhibit the impact that Tpm3.1 has on the actin filament (shown here for ATM1001 and in^21,25,26^ for other ATM compounds). We hypothesize that by altering the conformation of the actin filament, ATMs may be inhibiting the binding of other actin associated proteins. Previously we have shown that Tpm3.1 is able to recruit myosin II (MyoII) motors to actin stress fibres^6^. Furthermore, it has been demonstrated that MyoII motors are involved in insulin-stimulated glucose uptake^42,43^ and secretory granule exocytosis^35,36^, and we have shown that TR100 abrogates the recruitment of Tpm3.1 and MyoIIA to the cell cortex in response to insulin^23^. Therefore, the ATM drugs may be altering either the ability of the actin filament to recruit MyoII motors or MyoII function which in turn reduces the trafficking and/or fusion of GLUT4 vesicles and secretory granules with the plasma membrane leading to the inhibition of exocytosis.

## Conclusion

It has previously been shown that ATM compounds are able to specifically target Tpm3.1 in cultured cells^21^ and in cell-free *in vitro* systems^21,26^. The present results are a clear demonstration that the ATM compounds can impact their target Tpm3.1 in tissues of animals. This validates the usefulness of these ATM compounds to elucidate the function of specific Tpm filament populations in animal tissues. Gene knock-outs and knock-downs have enabled us to identify which functions of specific filament populations cannot be rescued or compensated in the whole animal. However, the development of anti-Tpm drugs allows us to directly examine the function of these filament populations in the whole body while minimising interference from compensating mechanisms which are likely to respond more slowly, if at all.

## Materials and Methods

### Antibodies and anti-tropomyosin compounds

The following primary antibodies were used: mouse monoclonal anti-Tpm3.1 antibody (2G10)^40^ and rabbit insulin antibody (C27C9, Cell Signalling). Alexa488-goat-anti-mouse (A11001), Alexa647-donkey-anti-mouse (A31571), Alexa488-donkey-anti-sheep (A11015), and Alexa647-donkey-anti-rabbit (A31573) secondary antibodies were used (Invitrogen). Atto-488 labelled phalloidin (AD 488–81) was obtained from ATTO-TEC (Siegen, Germany).

TR100 was the first-in-class compound anti-tropomyosin (ATM) designed to target tropomyosin isoform, Tpm3.1^21,26^. TR100 was sourced from SynMedChem and synthesised as previously described^21^. First generation small molecules were developed, using a computational docking approach, to target the sequence divergence at the C-terminus of the Tpm3.1 protein. Further modelling, docking and mapping of the 3-D steric and electronic motifs was used to generate a second-generation library of small molecules with the objective of improving the “drug like properties”, binding affinity and specificity for the C-terminus of Tpm3.1. ATM-1001 was identified as a “hit” from this second-generation library and was kindly supplied by Novogen Pty Ltd. The ATM compounds were formulated in 5% DMSO/30% Dexolve-7 (DavosPharma) adjusted to pH 6.8.

### Mice

Animal experiments were performed in accordance with UNSW Australia Animal Care and Ethics Committee approval and Australian National Health and Medical Research Council (NHMRC) guidelines. The Tpm3.1 knockout mouse line [B6-Tpm3^tm2(∆9d)Pgun^] was described previously^12^. All mice (10–12 week-old male) received standard rodent chow *ad libitum* (Rat Maintenance Diet, Gordon’s Specialty Feeds, Sydney, Australia) in a temperature controlled facility (22–24 °C) on a 12 h light (7 am)/dark (7 pm) cycle.

### Glucose and insulin tolerance tests (GTT and ITT)

The acute impact of the compounds was assessed by performing GTT and ITTs 1 h after ATM compound injection (*i.p*.). GTT and ITT were performed by injecting (*i.p*.) sterile glucose (1.5 or 2.0 g/kg BW) or insulin (1.5 U/kg BW; Actrapid, Novo Nordisk), respectively, into the drug-treated mice and glucose clearance measured as previously described^23^. To assess glucose-stimulated insulin secretion (GSIS), blood insulin was measured (Ultra Mouse Insulin ELISA kit; Crystal Chem) during a GTT.

### Insulin-stimulated glucose uptake in skeletal muscle

Glucose uptake in isolated EDL and soleus muscles was measured as described by Hansen *et al*.^44^. Muscles from anaesthetised mice (100 mg/kg BW ketamine and 10 mg kg/BW xylazine, *i.p*.) were incubated (30°C) in: (1) oxygenated (95%O_2_:5% CO_2_) Krebs-Henseleit buffer (KHB) (124 mM NaCl; 5 mM KCl; 3 mM CaCl_2_; 1.27 mM KH_2_PO_4_; 1.27 mM MgSO_4_.7H_2_O; 25 mM NaHCO_3_, pH 7.4) containing 0.1% BSA, 8 mM glucose, 32 mM mannitol for 1 h, then (2) KHB/BSA containing 4 mM pyruvate, 36 mM mannitol for 40 min and finally (3) KHB/BSA containing 4 mM 2-deoxyglucose (2DG) (with 55.5 kBq/mL [1-2-^3^H]-DG, Perkin Elmer), 36 mM mannitol (with 7.4 kBq/mL [^14^C]-mannitol), with or without insulin (14 nM) for 20 min. ATM drugs or vehicle was included in the last 2 incubation solutions (treatment time = 1 h). Muscles were then digested in 1 M KOH, neutralized (1 M HCl) and the extract counted for ^3^H and ^14^C radioactivity (Tri-Carb 2810TR β-scintillation counter, Perkin Elmer). The ^14^C-mannitol radioactivity was used to calculate muscle extracellular volume. Extracellular ^3^H-2DG was subtracted from total ^3^H-2DG to determine intracellular uptake^44^.

### Western blots

Muscle was solubilised in radioimmunoprecipitation assay buffer [(20 mM Tris; 150 mM NaCl; 1% Nonidet P-40; 0.5% sodium deoxycholate; 1 mM EDTA; 0.1% SDS; 1 Complete EDTA-free protease inhibitor tablet (Roche); 1 PhosSTOP phosphatase inhibitor tablet (Roche)] and protein (20 μg) run on SDS PAGE (12.5% resolving gel) gels and proteins transferred to polyvinylidene fluoride (PVDF) membranes (Merck Millipore). Membranes were blocked for 2 h with 1% BSA in Tris-buffered saline (100 mM Tris-HCl pH 7.5, 150 mM NaCl, 0.1% Tween 20; TBST) at room temperature (RT). Blots were incubated overnight at RT with primary antibodies [rabbit-poly clonal Akt (1:1000) and pAkt (Ser473) (1:1000), Cell Signaling Technology, Inc; rabbit-poly clonal pan 14-3-3 (1:1000), Santa Cruz Biotechnology]. Blots were then washed with TBST and incubated with donkey-anti-rabbit/horse radish peroxidase (1:10 000 dilution in TBST/2% skim milk; Jackson ImmunoResearch Laboratories) for 2 h AT. Blots were then washed with TBST and incubated with Luminata Cresecendo chemiluminescent HRP reagent (Merck Millipore) and then imaged using the ChemiDoc MP system (BioRad). Densitometry values were corrected for the loading control (pan-14-3-3). The results were then expressed as a ratio to an internal standard (insulin-stimulated muscle) run on each gel.

### Glucose-stimulated insulin secretion in isolated islets

Mice were anaesthetized with ketamine/xylazine and their islets isolated as described by Chan *et al*.^45^. Groups of five islets, with three to four replicates per animal, were incubated at 37 °C in Krebs-Ringer HEPES buffer^45^ containing ATM compound or vehicle and 5 or 20 mM glucose for 1 h. Insulin was measured in the incubation to measure insulin secretion.

### Glucose-stimulated insulin secretion and staining of MIN6 cells

MIN6 β-cells were cultured as described^46^. For insulin secretion experiments, cells were seeded in 24-well plates (2 × 10^5^cells/well), incubated at 37°C in KRH buffer for 1 h, then 2.8 or 25 mM glucose KRH for 1 h. ATM compound or vehicle was included in both incubations. Insulin was measured in the final incubation buffer to assess impact on insulin secretion. Data was obtained from 5–6 independent experiments.

To assess the impacts on the actin cytoskeleton, MIN6 cells were seeded onto cover slips (No.1; Menzel Glass, MENCSC191GP; Pacific Laboratory Products, Blackburn, VIC, Australia), cultured for 48 h and treated with ATM compounds and glucose as described for insulin secretion. Cells were then fixed with 4% formaldehyde (15 min at RT) at various times after glucose stimulation (10–30 min), permeabilized with 0.1% Triton X-100 (30 min), stained with 10 nM Atto488-phalloidin (containing 2% BSA) for 1 h, washed in PBS and then mounted with Aqua PolyMount (Polysciences). Cells were imaged on a Nikon A1 confocal using a 100X Plan Apo 1.4 100X Oil objective.

### Immunofluorescent staining of mouse pancreas

Mouse pancreas was fixed in 2% PFA (2–3 h at 4°C), infused with 15% sucrose overnight, embedded in Tissue-Tek and frozen in liquid nitrogen cooled isopentane. Sections (10 μm) were cut (Leica CM1950 cryostat), treated with blocking buffer (5% goat serum, 5% FBS, 1% BSA in PBS, 1–2 h at RT) and stained with anti-Tpm3.1 (2G10, 1:25) (Schevzov *et al*.^40^) and/or (C27C9; 1:800) insulin antibodies for 12 h at RT. Sections were then stained with AlexaFluor488 secondary antibody (1:750) or AlexaFlour647 secondary antibody combined with Atto488-phalloidin (1:250) for 1–2 h at RT. Sections were imaged on an Olympus FV1200 confocal microscope.

### Statistical analysis

Data was analysed by Student’s T-test (2 group comparisons) or by ANOVA followed by Tukey’s multiple group comparison test (Graphpad Prism v6.05) (*p* < 0.05 was considered statistically significant). Data are expressed as mean ± SEM.

## Electronic supplementary material

Supplemental Material
