# Supplementary material for: On-target action of anti-tropomyosin drugs regulates glucose metabolism
Source: Sci Rep. 2018 Mar 15;8:4604. doi: 10.1038/s41598-018-22946-x (PMC5854615; doi:10.1038/s41598-018-22946-x)
Supplement: Supplementary file 1 — Supplemental Material [file 41598_2018_22946_MOESM1_ESM.pdf]

Supplemental Material

On-target action of anti-tropomyosin drugs regulates glucose metabolism

Anthony J. Kee, Jayshan Chagan, Jeng Yie Chan, Nicole S. Bryce, Christine A. Lucas, Jun Zeng, Jeff Hook, Herbert Treutlein, D. Ross Laybutt, Justine R. Stehn, Peter W. Gunning and Edna C. Hardeman

SUPPLEMENTAL METHODS

Molecular Modeling of ATM-1001 binding to Tpm3.1

For the prediction and modeling of the binding mode of ATM-1001 to Tpm3.1 we applied a method similar to the one described previously[1]. A homology model of Tpm3.1 was first built based on the solution NMR structure of the junction between tropomyosin molecules[2]. The coordinates for the NMR structures were obtained from the PDB databank (PDB id 2g9j)[3] and a representative structure was chosen out of the 10 NMR models as the template structure. The homology model was created using SWISS-MODEL[4], however, the helical conformation and side chain packing were optimized using a simulated annealing procedure based on Smith *et al.*[5] using NAMD2[6].

The binding site available for ATM-1001 on the Tpm3.1 dimer was examined using a "Multiple Copy Simultaneous Search" (MCSS) method[7] where the preferred positions of 400 indole rings representing the scaffold of ATM-1001 were determined computationally around the C-terminus of Tpm3.1. Ten indole fragment clusters were found to be located inside the cavity of the C-terminus formed between two helices. These clusters then served as guidance for docking of ATM-1001 to the C-terminus. Our software "Qu-Cbit" (version 3.01, MedChemSoft Solutions, Melbourne, Australia) was used for the MCSS and docking calculations. Four conformations were selected followed by further refinement at quantum mechanical level. The QM calculations applied the GAMESS program (http://www.msg.ameslab.gov/gamess) to a system consisting of the ATM-1001 scaffold and ten Tpm3.1 residues surrounding the ligand. The QM calculations optimized the geometry of ATM-1001 with the protein residues kept fixed. Density Functional Theory method was applied using the B3LYP functional[8,9] and the 6-31G(d) basis set[10]. The conformation with the lowest total energy was selected as the best binding mode of the ATM-1001 scaffold. Using the optimized structure of the scaffold, the missing parts of ATM-1001 were added, followed by QM refinement of the geometry of the full ATM-1001 with the fixed residues of Tpm3.1.

ATM-1001 Tpm3.1 filament disruption assay

Mouse embryonic fibroblasts (MEF) were isolated and cultured as previously described [11]. MEFs were seeded (5 x 10^3/well) into 8-well chamber slides (NUNC, Thermofisher Scientific) and treated with d 5 µM ATM-1001 for 24h using DMSO as vehicle control. Twenty-four hours post treatment, cells were fixed with 4% paraformaldehyde (PBS), permeabilized with Triton-X-100 and stained with γ9d (1:100) followed by 488-conjugated sheep secondary antibody (1:1000) and DAPI to visualize the Tpm3.1 containing filament bundles and the nucleus, respectively. Single plane images were obtained on the Zeiss epifluorescent Axioscope microscope using a 20x objective. Six fields of view per condition were imaged. Changes in the number and length of Tpm3.1 filament bundles within each cell were quantitated using a linear feature detection algorithm developed by the Commonwealth Scientific and Industrial Research Organisation (CSIRO) as described previously[3].

Pyrene–actin depolymerization assay

Actin depolymerisation assays were performed as described by Bonello *et al.* [12]. Prior to the addition to polymerized pyrene labelled F-actin (3 µM), Tpm3.1 dimers (5 µM) were incubated (10 min, RT) with 50 µM of ATM-1001. The F-actin/Tpm3.1 ± ATM-1001 was incubated 30 min RT and samples were then transferred to a "black walled" 96 well plate. Duplicate samples were diluted 12-fold using an F-actin polymerization buffer (100 mM NaCl; 10 mM Tris HCl pH 7; 2 mM $MgCl_2$; 1 mM EGTA; 0.2 mM ATP; 0.5 mM DTT) and the polymerization rates of F-actin alone, F-actin/Tpm3.1 and the F-actin/Tpm3.1/ATM-1001 filament complex were

measured using a Perkin Elmer EnSpire fluorescence plate reader (407 nm) at 30 second intervals for 60 min. (RT). Data was normalized to the initial fluorescence value and polymerization curves of duplicate samples were fitted to a linear regression model using GraphPad Prism 6. Percent polymerization was determined at t_{60} as a function of t_0 (100%) for n = 3 independent experiments.

Plasma clearance and tissue distribution of ATM-1001

Mice were injected (*i.p.*) with ATM-1001 (40 mg/kg BW) and blood (cardiac puncture) and tissues taken at 0.5, 1, 2, 4 and 8 h after injection. Plasma and tissues were sent on dry ice to Jubilant Biosys (Bangalore, India) for analysis. Briefly, tissues were homogenized in five volumes of PBS (pH 7.4) on ice using a Miccra D9 homogenizer. To 50 µL of plasma and tissue homogenates, 0.2 mL of acetonitrile was added, vortexed for 5 min and centrifuged at 10000 x g, 5 min. at 4°C. Two microliters of the supernatant was analysed for ATM1001 content by LC-MS/MS. The LC-MS-MS system consisted of a Shimadzu LC-20 AD Series HPLC system and a AB Sciex API-4000 Q-trap mass spectrometer, equipped with a Turboionspray™ interface at 500°C and 5000 V ion spray voltage used in positive ion mode. Quantitation of ATM-1001 was achieved by monitoring the precursor (Q1)/product (Q3) ions at *m/z* 469/279 and 515/307, respectively. The analytical data were processed by Analyst software (version 1.6.3).

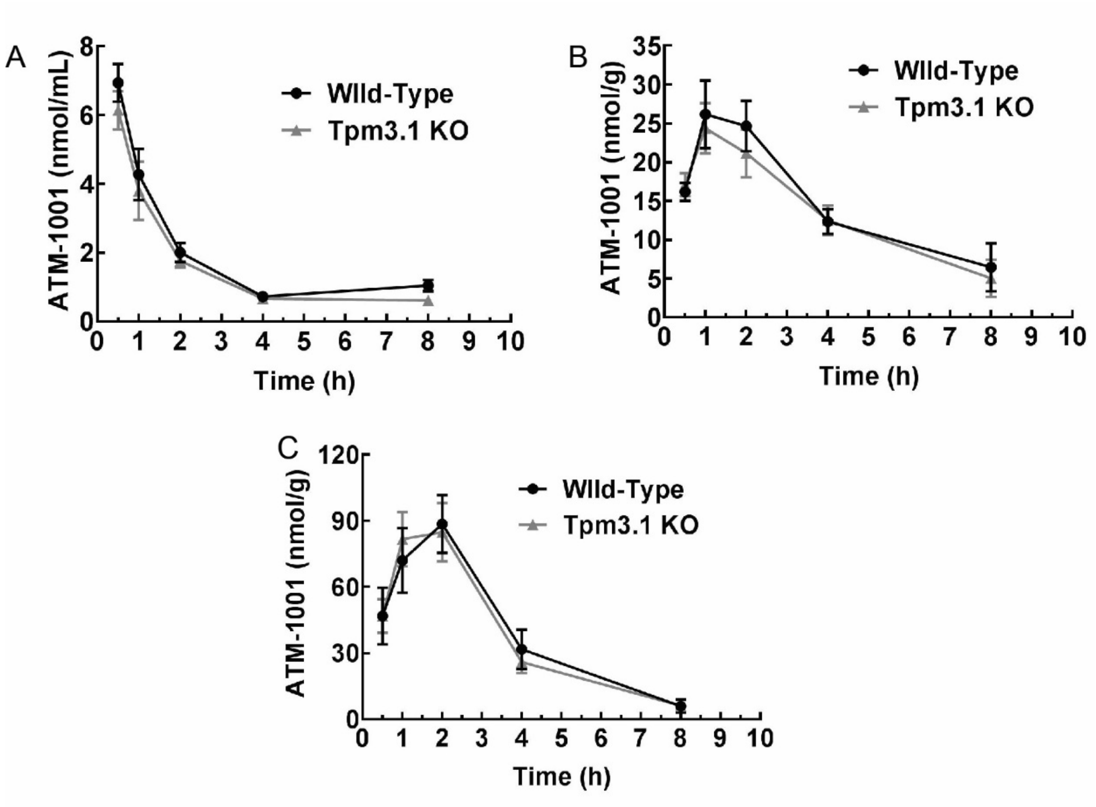

Figure S1: Plasma clearance and tissue distribution of ATM-1001 is similar in wild-type and Tpm3.1 knock out (KO) mice. Concentration of ATM-1001 in (A) plasma, (B) skeletal muscle (quadriceps) and (C) pancreas of wild-type and Tpm3.1 knock-out (KO) mice following ATM-1001 injection (40 mg/kg BW, *i.p.*). Data is mean ± SEM for n = 4-5/group.

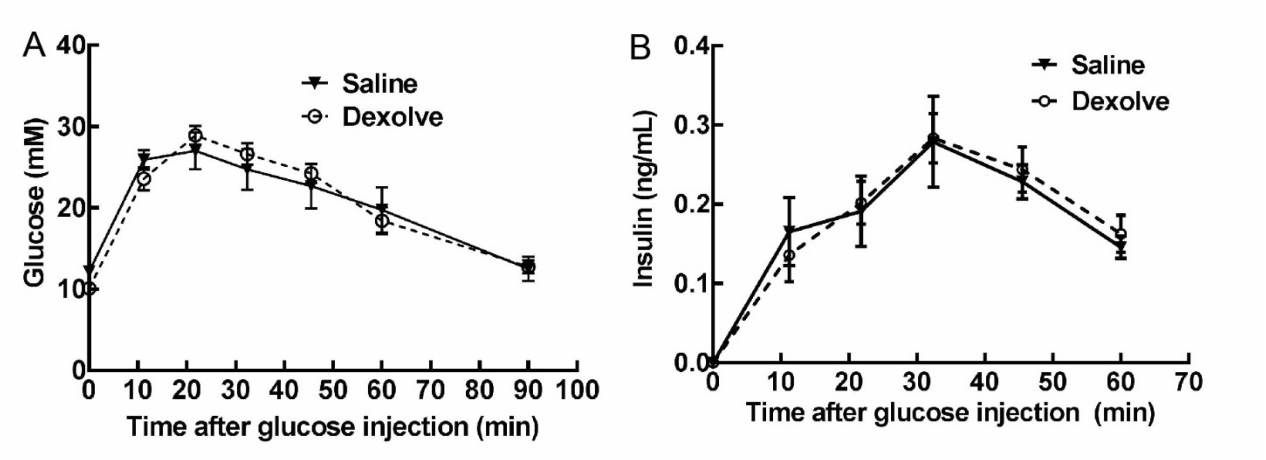

Figure S2: ATM drug vehicle (Dexolve) has no impact on glucose clearance or glucose-stimulated insulin secretion. (A) Blood glucose and (B) insulin levels during a glucose tolerance test (GTT; 1.5 g glucose/kg BW, *i.p.*), 1h after Dexolve injection in wild-type mice. The amount of Dexolve administered (3 g/kg BW, *i.p.*) is equivalent to that delivered with the ATM drugs. Data represents mean ± SEM for n = 5 mice/group.

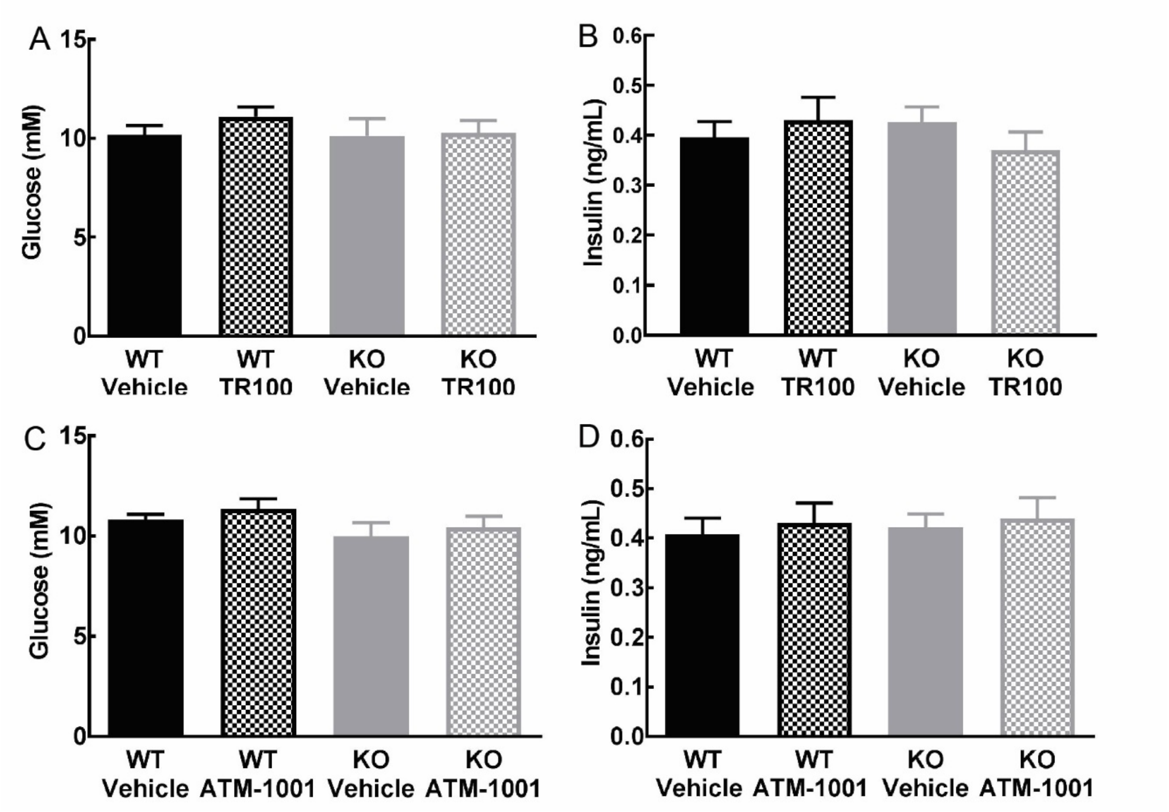

Figure S3: Impact of ATM drugs on fasting glucose and insulin. (A, C) Fasting (6h) blood glucose and (B, D) and insulin 1h after TR100 (A , B) or ATM-1001 (C, D) injection (40 mg/kg BW, *i.p.*) in wild-type (WT) and Tpm3.1 knock-out (KO) mice (n = 6-8/group). Results are shown as mean ± SEM and the results of statistical analysis (ANOVA with post-hoc Tukeys' multiple comparison test) are indicated: ns P > 0.05, * P < 0.05, ** P < 0.01.

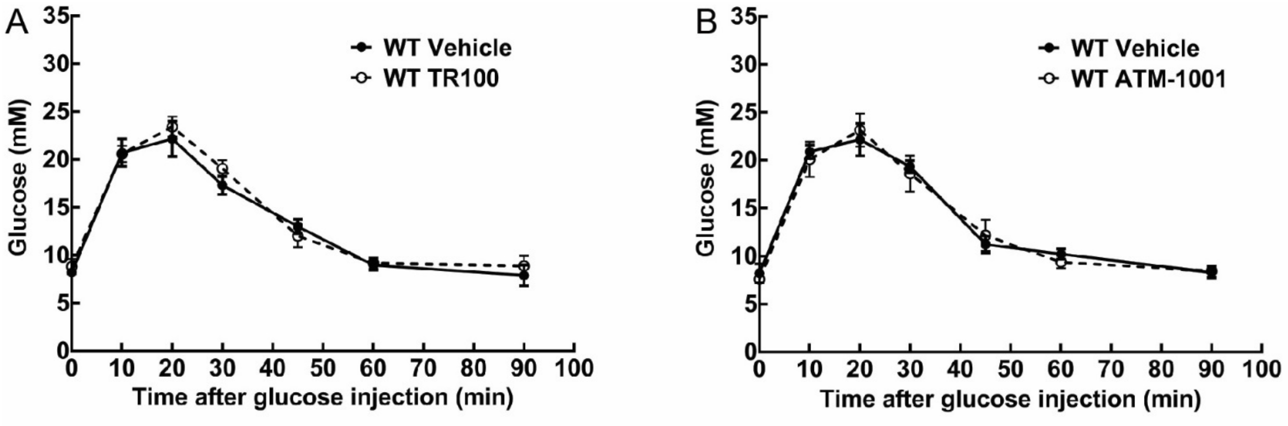

Figure S4: TR100 and ATM-1001 do not have prolonged impact on glucose clearance. Glucose tolerance test (GTT; 2 g glucose/kg BW, *i.p.*) performed 8h after (A) TR100 or (B) ATM1001 injection (40 mg/kg BW, *i.p.*) in wild-type (WT) mice. Results are shown as mean ± SEM for n = 5-7/group.

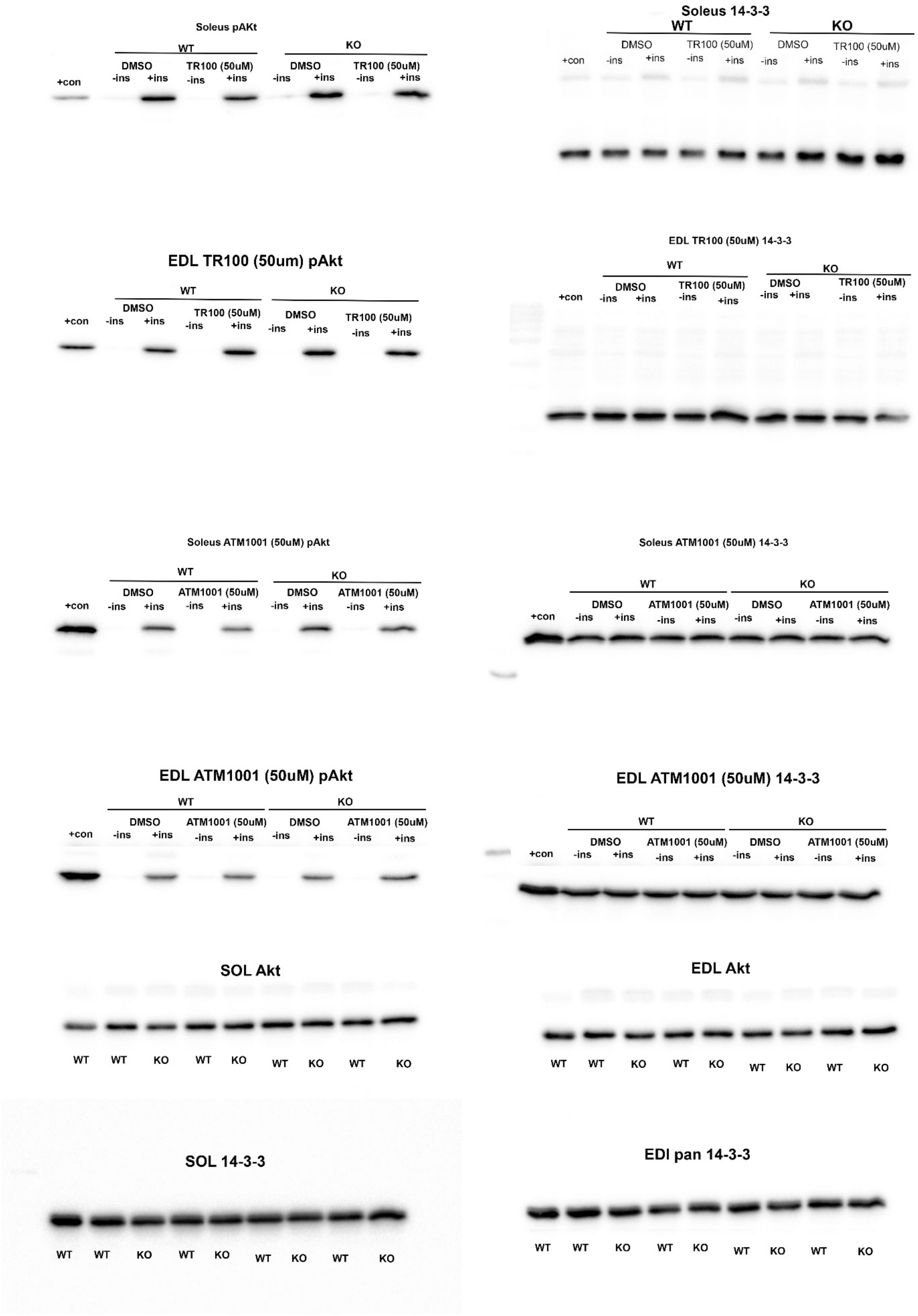

Figure S5: Original unedited Akt Western blots.

References

1 Stehn, J. R. *et al.* A novel class of anticancer compounds target the actin cytoskeleton in tumor cells. *Cancer Res.* **73**, 5169-5182 (2013).

2 Greenfield, N. J., Kotlyanskaya, L. & Hitchcock-DeGregori, S. E. Structure of the N terminus of a nonmuscle alpha-tropomyosin in complex with the C terminus: implications for actin binding. *Biochemistry* **48**, 1272-1283 (2009).

3 Berman, H. M. The Protein Data Bank: a historical perspective. *Acta Crystallogr. A* **64**, 88-95 (2008).

4 Arnold, K., Bordoli, L., Kopp, J. & Schwede, T. The SWISS-MODEL workspace: a web-based environment for protein structure homology modelling. *Bioinformatics* **22**, 195-201 (2006).

5 Smith, D. K. *et al.* Homology modelling and 1H NMR studies of human leukaemia inhibitory factor. *FEBS Lett.* **350**, 275-280 (1994).

6 Phillips, J. C. *et al.* Scalable molecular dynamics with NAMD. *J. Comput. Chem.* **26**, 1781-1802 (2005).

7 Zeng, J. & Treutlein, H. R. A method for computational combinatorial peptide design of inhibitors of Ras protein. *Protein Eng.* **12**, 457-468 (1999).

8 Becke, A. D. Density-functional exchange-energy approximation with correct asymptotic behavior. *Phys. Rev. A Gen. Phys.* **38**, 3098-3100 (1988).

9 Lee, C., Yang, W. & Parr, R. G. Development of the Colle-Salvetti correlation-energy formula into a functional of the electron density. *Phys. Re.v B Condens. Matter* **37**, 785-789 (1988).

10 Krishnan, R., Binkley, J. S., Seeger, R. & Pople, J. A. Self-consistent molecular orbital methods. XX. A basis set for correlated wave functions. *J. Chem. Phys.* **72**, 650-654 (1980).

11 Schevzov, G. *et al.* Tissue-specific tropomyosin isoform composition. *J. Histochem. Cytochem.* **53**, 557-570 (2005).

12 Bonello, T. T. *et al.* A small molecule inhibitor of tropomyosin dissociates actin binding from tropomyosin-directed regulation of actin dynamics. *Sci Rep*, doi:10.1038/srep19816 (2016).

13 Kee, A. J. *et al.* An actin filament population defined by the tropomyosin Tpm3.1 regulates glucose uptake. *Traffic* **16**, 691–711 (2015).
